# Use of Flexible Cystoscopy to Insert a Foley Catheter over a Guide Wire in Spinal Cord Injury Patients: Special Precautions to be Observed

**DOI:** 10.1155/2011/538750

**Published:** 2011-10-26

**Authors:** Subramanian Vaidyanathan, Bakul Soni, Gurpreet Singh, Peter Hughes, Tun Oo

**Affiliations:** ^1^North West Regional Spinal Injuries Centre, Southport and Formby District General Hospital, Town Lane, Southport PR8 6PN, UK; ^2^Department of Radiology, Southport and Formby District General Hospital, Southport PR8 6PN, UK

## Abstract

When urethral catheterisation is difficult or impossible in spinal cord injury patients, flexible cystoscopy and urethral catheterisation over a guide wire can be performed on the bedside, thus obviating the need for emergency suprapubic cystostomy. Spinal cord injury patients, who undergo flexible cystoscopy and urethral catheterisation over a guide wire, may develop potentially serious complications. (1) Persons with lesion above T-6 are susceptible to develop autonomic dysreflexia during cystoscopy and urethral catheterisation over a guide wire; nifedipine 5–10 milligrams may be administered sublingually just prior to the procedure to prevent autonomic dysreflexia. (2) Spinal cord injury patients are at increased risk for getting urine infections as compared to able-bodied individuals. Therefore, antibiotics should be given to patients who get haematuria or urethral bleeding following urethral catheterisation over a guide wire. (3) Some spinal cord injury patients may have a small capacity bladder; in these patients, the guide wire, which is introduced into the urinary bladder, may fold upon itself with the tip of guide wire entering the urethra. If this complication is not recognised and a catheter is inserted over the guide wire, the Foley catheter will then be misplaced in urethra despite using cystoscopy and guide wire.

## 1. Introduction

Many patients with spinal cord injury and neuropathic bladder dysfunction require long-term indwelling catheter drainage or intermittent catheterisations. Repeated urethral catheterisation exposes these patients to the risk of urethral trauma. Sometimes, urethral catheterisation, especially when performed by an inexperienced health professional, may cause injury to urethra and create a false passage. Urethral trauma can lead to profuse bleeding per urethra. When a false passage is created by traumatic catheterisation, subsequent urethral catheterisations may be difficult or even impossible. In such an instance, it is advisable to perform flexible cystoscopy instead of trying urethral catheterisation blindly. By performing flexible cystoscopy, the urologist is able to assess the extent of urethral trauma, exact site of urethral injury, and direction and depth of urethral false passage [1, 2].

## 2. Technique of Inserting a Catheter per Urethra over a Guide Wire in Spinal Cord Injury Patients

### 2.1. Indications

1. Trauma while inserting a catheter per urethra leading to significant bleeding.
2. Total inability to pass a catheter per urethra either for intermittent catheterisation or for indwelling purpose.
3. Difficulty in passing a catheter per urethra.
4. Patients with established urethral false passage.

### 2.2. Technique

Details of the procedure are explained to the spinal cord injury patient, and then informed consent is obtained. Cystoscopy is performed while the patient lies comfortably supine on his/her bed. A video cystoscope or a conventional flexible cystoscope is inserted into the urinary bladder. If there is lot of bleeding, which obscures the vision, the bag of irrigating fluid (0.9% sodium chloride solution) is squeezed for a few seconds to achieve clear vision and, thereby, enable safe passage of cystoscope through the urethra. A 0.032′′ diameter, 145 cm long, fixed core straight, guide wire (TSF-32-145, Cook Inc. Bloomington, Ind, USA) is inserted through flexible cystoscope. The tip of guide wire should be seen inside the bladder. The cystoscope is then withdrawn while advancing the guide wire further thus ensuring that the tip of guide wire stays inside the bladder. Then a Foley catheter, which has open proximal end, is threaded over the guide wire. When the Foley catheter is inserted into the urinary bladder, urine will start draining from the Foley catheter. After inflating the balloon of Foley catheter, the guide wire is removed. Thus flexible cystoscopy ensures safe insertion of a Foley catheter into the urinary bladder in patients with urethral trauma or false passages.

### 2.3. Followup

During followup, usually after four to six weeks, the urethral catheter is changed using a guide wire. A 0.032′′ guide wire is inserted through the Foley catheter which has open proximal end. Then the balloon of Foley catheter is deflated and the catheter is withdrawn. A new Foley catheter is inserted over the guide wire. If insertion of guide wire is not possible because of partial or total blockage of the lumen of Foley catheter, the blocked catheter is removed and flexible cystoscopy is performed. A new Foley catheter is inserted over a guide wire introduced under vision through flexible cystoscope.

## 3. Special Considerations While Performing Flexible Cystoscopy in Spinal Cord Injury Patients

### 3.1. Adaptations Required for Insertion of Foley Catheter over a Guide Wire

Foley catheters with proximal open end are commercially available. We have been using Folysil catheter, size 16 French, 10 mL balloon (Reference AA7416, Coloplast Ltd, Peterborough PE2 6BR, UK) for spinal cord injury patients, who require insertion of a catheter over a guide wire. Use of Folysil catheter obviates the need to make a hole at the proximal end. If Folysil catheter is not available, a hole may be made at the proximal end of a conventional Foley catheter with the help of a Foley Punch, the tip of catheter may be cut longitudinally with a scalpel [3], or a 14-gauge (Brown) intravenous cannula needle can be threaded through the side drainage hole of the Foley catheter [4].

### 3.2. Autonomic Dysreflexia

Patients with spinal injury above midthoracic level (T-6) may develop autonomic dysreflexia during flexible cystoscopy. Spinal cord injury patients may develop severe, pounding headache and sweating. Therefore, vital signs such as blood pressure, heart rate, and oxygen saturation should be monitored during flexible cystoscopy in spinal cord injury patients. If a patient develops headache or an increase in blood pressure is observed during cystoscopy, nifedipine should be administered sublingually. We administer five milligrams of nifedipine, and another dose of 5 mg is repeated if symptoms do not subside within five minutes. In order to reduce the chances of autonomic dysreflexia, distension of urinary bladder should be avoided during cystoscopy. When urethral catheter is inserted successfully into the urinary bladder and urine is drained, symptoms of autonomic dysreflexia will subside rapidly.

### 3.3. Urine Infection and Septicaemia

Patients with spinal cord injury and neuropathic bladder often exhibit asymptomatic bacteriuria. Following flexible cystoscopy, these patients are at risk for getting urine infection. Very rarely, a patient may develop septicaemia after undergoing flexible cystoscopy. Spinal cord injury patients, who develop bleeding from urethra or who pass blood-stained urine following flexible cystoscopy, are at increased risk for developing bacteraemia and septicaemia following insertion of a catheter per urethra over a guide wire. Therefore, all spinal cord injury patients, who undergo flexible cystoscopy, should be observed at least for two hours following the procedure.

### 3.4. Should Antibiotics Be Given to Patients Who Undergo Insertion of Foley Catheter over a Guide Wire?

In spinal cord injury patients, who undergo flexible cystoscopy and change of urethral catheter over a guide wire *as an elective procedure,* routine administration of antibiotic may not be necessary. However, if a spinal cord injury patient develops even mild degree of haematuria after insertion of catheter over a guide wire, it will be prudent to administer 160 mg of gentamicin intramuscularly. In spinal cord injury patients with urethral trauma and bleeding per urethra, it is advisable to insert a Venflon for venous access and administer gentamicin 240 milligrams intravenously before performing flexible cystoscopy.

In spinal cord injury patients, who tend to develop urine infection following flexible cystoscopy, we send a sample of urine for microbiology five to seven days before performing flexible cystoscopy. In this subgroup of spinal cord injury patients, who have innate tendency to develop urine infections after change of urethral catheter, the appropriate antibiotic based on the antibiotic sensitivity report of urine sample is administered just prior to flexible cystoscopy.

## 4. Potential Complications That Can Occur during Insertion of a Catheter over a Guide Wire in Spinal Cord Injury Patients with Small Capacity Bladder

### 4.1. Incorrect Positioning of Foley Catheter despite Using a Guide Wire for Insertion

**Figure 1:**
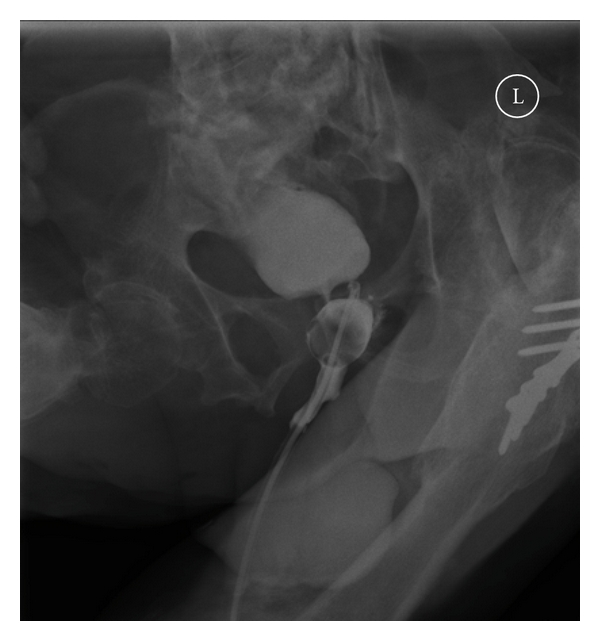
Cystogram showed the tip of catheter to be located outside the bladder in the urethral false passage.

**Figure 2:**
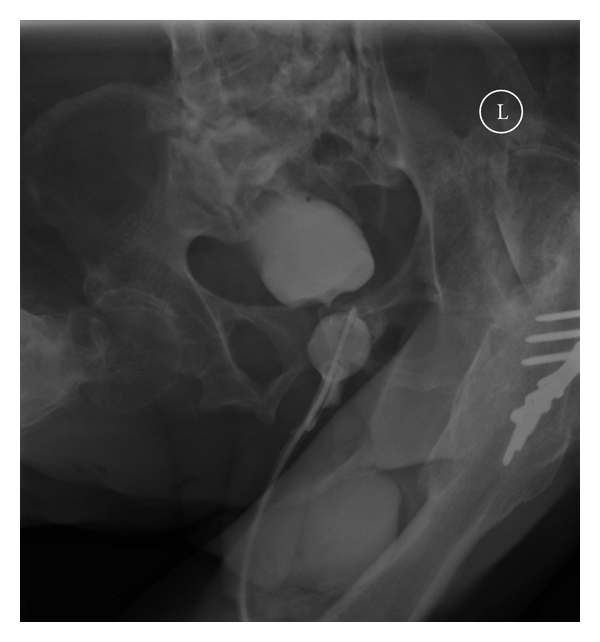
Two mL of contrast was then injected through balloon channel of Foley catheter to visualise the balloon. The balloon could be seen lying in the urethra.

Spinal cord injury patients may develop a small capacity urinary bladder after prolonged indwelling catheter drainage. Similarly, patients with low-compliant bladder are likely to exhibit small capacity bladder. In these patients, extra care should be observed during insertion of a guide wire. The guide wire may fold upon itself, and the tip of guide wire may find its way through bladder neck into the proximal urethra or even the false passage in urethra. If a catheter is then inserted over the misplaced guide wire, the tip of catheter will be located outside the urinary bladder and the balloon of Foley catheter will be located in the urethra. Then the catheter will not drain urine and the patient will experience leakage of urine around the misplaced catheter. Such a problem was encountered recently while changing urethral catheter over a guide wire in a 47-year-old male patient with paraplegia and urethral false passage. Following flexible cystoscopy and urethral catheterisation over a guide wire, the catheter failed to drain urine and urine was leaking around the catheter. Therefore, cystogram was performed (Figures 1 and 2). Cystogram showed that the tip of Foley catheter was lying outside the bladder. The Foley balloon was located in the proximal urethra. 

When a Foley catheter fails to drain urine and urine is leaking around the catheter, position of the balloon can be verified using ultrasound on the bedside. Ultrasound examination is preferable to cystourethrography, as bedside ultrasound avoids radiation as well as unnecessary transfer of the patient with spinal cord injury to radiology department.

Urologists should be vigilant to recognise this complication while withdrawing the flexible cystoscope after insertion of a guide wire. Should the guide wire fold upon itself and enter the urethra, the guide wire must be withdrawn and repositioned so that the tip of the guide wire is located well within the urinary bladder.

### 4.2. Perforation of the Bladder by the Tip of Guide Wire

The wall of urinary bladder may be inflamed and friable in some spinal cord injury patients. In these patients, the guide wire may perforate the bladder, especially if the guide wire is inserted forcefully and rapidly into the bladder. Very rarely, the stiff end of guide wire may be inserted if the guide wire had been packaged incorrectly. On rare occasions, the flexible tip of guide wire may enter the ureteric orifice, particularly in spinal cord injury patients with vesicoureteric reflux and gaping ureteric orifice. The urologist should check the location of the tip of guide wire before withdrawing the flexible cystoscope. Thus this technique of insertion of a catheter over a guide wire in a spinal cord injury patient warrants concentration and full attention during the entire procedure.

## 5. Who Should Perform Urethral Catheterisation over a Guide Wire in Spinal Cord Injury Patients?

Flexible cystoscopy and urethral catheterisation over a guide wire in spinal cord injury patients should be carried out by an experienced doctor. Even in the hands of a senior doctor, this procedure may prove to be difficult. When the first attempt to insert a catheter over a guide wire is unsuccessful, the doctor should seek help from a senior urologist immediately. The aim should be to perform this procedure safely without producing any complication.
